# Hydroxy Pentacyclic Triterpene Acid, Kaempferol, Inhibits the Human 5-Hydroxytryptamine Type 3A Receptor Activity

**DOI:** 10.3390/ijms23010544

**Published:** 2022-01-04

**Authors:** Shinhui Lee, Hee-Soo Seol, Sanung Eom, Jaeeun Lee, Chaelin Kim, Jong-Hwan Park, Tae-Hwan Kim, Junho H. Lee

**Affiliations:** 1 Department of Biotechnology, Chonnam National University, Gwangju 61186, Korea; dltlstn39@gmail.com (S.L.); yeomself2355@gmail.com (S.E.); je_lee@hugel.co.kr (J.L.); cl215@biometro.net (C.K.); 2 College of Veterinary Medicine and Animal Medical Institute, Chonnam National University, Gwangju 61886, Korea; heesoo@hugel.co.kr (H.-S.S.); jonpark@jnu.ac.kr (J.-H.P.); 3 Department of Animal Science, Chonnam National University, Gwangju 61886, Korea; grassl@chonnam.ac.kr

**Keywords:** serotonin receptor, Kaempferol, hydroxy pentacyclic, neuroprotective, TEVC

## Abstract

Monoamine serotonin is a major neurotransmitter that acts on a wide range of central nervous system and peripheral nervous system functions and is known to have a role in various processes. Recently, it has been found that 5-HT is involved in cognitive and memory functions through interaction with cholinergic pathways. The natural flavonoid kaempferol (KAE) extracted from *Cudrania tricuspidata* is a secondary metabolite of the plant. Recently studies have confirmed that KAE possesses a neuroprotective effect because of its strong antioxidant activity. It has been confirmed that KAE is involved in the serotonergic pathway through an in vivo test. However, these results need to be confirmed at the molecular level, because the exact mechanism that is involved in such effects of KAE has not yet been elucidated. Therefore, the objective of this study is to confirm the interaction of KAE with 5-HT_3A_ through electrophysiological studies at the molecular level using KAE extracted from *Cudrania tricuspidata*. This study confirmed the interaction between 5-HT_3A_ and KAE at the molecular level. KAE inhibited 5-HT_3A_ receptors in a concentration-dependent and voltage-independent manner. Site-directed mutagenesis and molecular-docking studies confirmed that the binding sites D177 and F199 are the major binding sites of human 5-HT_3A_ receptors of KAE.

## 1. Introduction

Monoamine serotonin (5-HT, 5-hydroxytryptamine) is a major neurotransmitter that acts on a wide range of central nervous system (CNS) and peripheral nervous system (PNS) functions. It is known to have a role in various processes such as immunity, gastrointestinal physiology [1], activity rhythms, sexual behavior, and emotional state [2]. Recently, it has been found that 5-HT is involved in cognitive and memory functions through interaction with cholinergic pathways [3]. Receptors that are activated by serotonin are divided into 5-HT1–5-HT7 classes [4] with various subtypes [1]. While other 5-HT receptors (5-HTRs) are G-protein coupled receptors (GPCRs) that serve as second messengers [5], 5-HT3 receptors are ligand-gated cation channels activated by binding of ligand for rapid synaptic neurotransmission [6].

5-HT_3A_ is a representative receptor of 5-HT_3_Rs comprising five A subunits to form a homomeric pentamer with subunits surrounding the central ion-permeable pore [7]. The ligand-binding site of 5-HT_3A_ is a ‘conserved aromatic cage’ formed by W63, Y126, W156, Y207, and F199 at the A+A interaction surface of the extracellular domain (ECD) [8]. Ligand binding causes a conformational change [8] and opens the central ion pore that allows cations to pass through the central pore [9]. It is known that 5-HT_3A_ expressed in pre-synaptic neurons can induce calcium influx, which plays a critical role in the release of neurotransmitters. When they are expressed in post-synaptic neurons, they can induce K^+^ and Na^+^-mediated rapid depolarization [10]. This process can convert chemical signals into electrical signals [11].

*Cudrania tricuspidata* (silkworm thorn), a regional plant containing several flavonoids and xanthones, has been used in traditional medicine. It possesses anti-cancer effects against various tumors [12,13]. The natural flavonoid kaempferol (KAE), extracted from *Cudrania tricuspidata,* is a secondary metabolite of the plant. It is well known for its anti-cancer and anti-inflammatory activities [14]. KAE has a yellow color with a diphenyl propane structure (C6-C3-C6) [15]. Recently studies have confirmed that KAE possesses a neuroprotective effect [16] because of its strong antioxidant activity [17]. KAE can inhibit xanthine oxidase that generates reactive oxygen species (ROS) [18] and increase activities of antioxidant enzymes (superoxide mutase, catalase, and heme oxygenase-1). It can also inhibit lipid peroxidation induced by ROS [19]. It is known that excessive accumulation of metal ions such as Fe^3+^ and Al^3+^ can induce neurodegenerative diseases [20]. However, KAE has the ability to remove ferrous and cuprous ions as a chelator.

It has been confirmed that KAE is involved in the serotonergic pathway through an in vivo test [21]. However, these results need to be confirmed at the molecular level because the exact mechanism involved in such effects of KAE has not yet been elucidated. Therefore, the objective of this study is to confirm the interaction of KAE with 5-HT_3A_ through electrophysiological studies at the molecular level using KAE extracted from *Cudrania tricuspidata*. This study confirmed the interaction between 5-HT_3A_ and KAE at the molecular level. KAE inhibited 5-HT_3A_ receptors in a concentration-dependent and voltage-independent manner.

## 2. Results

### 2.1. Kaempferol Inhibits Human 5-HT_3A_ Receptor in a Reversible and Concentration-Dependent Manner

**Figure 1:**
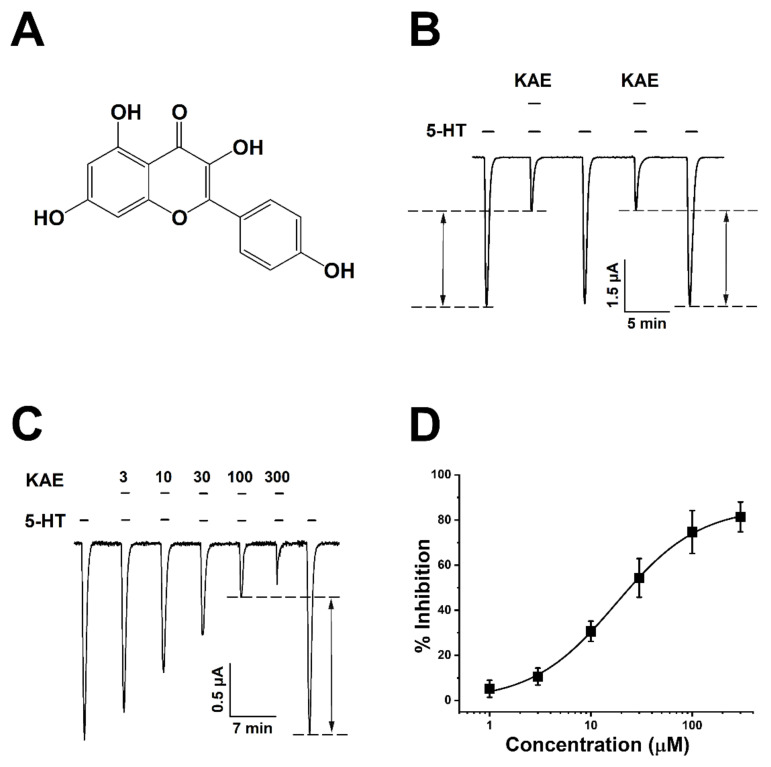
Chemical structure of Kaempferol (KAE) and activation manner confirmed by application to *Xenopus* oocytes expressing human 5-HT_3A_ receptor. (**A**) Structure of KAE. (**B**–**D**) Confirmation of KAE’s inhibition on *I*_5-HT_ using a two-electrode voltage clamp. (**B**) Confirmation of the inhibitory manner of KAE on *I*_5-HT_. Black bars indicate applied serotonin and KAE. Applied concentration of serotonin and KAE was 100 μM. The black dot line indicated in the figure represents the inward current changed when KAE was applied with the control ligand (5-HT, 100 μM) (*n* = 6–8 oocytes from four different frogs). (**C**) Confirmation of concentration-dependent inhibitory manner of KAE on *I*_5-HT_. Black bar indicates the application serotonin and KAE. The applied concentration of serotonin was fixed at 100 μM and the applied concentration of KAE is indicated in the figure (3–300 μM) (*n* = 7–9 oocytes from four different frogs). (**D**) *I*_5-HT_ inhibitory current according to the concentration-specific application of KAE is shown as a normalized curve. The inhibition percentage for human 5-HT_3A_ was fitted to the Hill equation. The *x*-axis shows the applied concentration of KAE and the *y*-axis shows the inhibition percentage of KAE on 5-HT_3A_. Each point represents the inhibition percentage, error value, and mean ± SEM (*n* = 7–9 oocytes from four different frogs) according to statistical analysis. All experiments were carried out at −80 mV voltage-clamp holding potential and the rate of application of the agent was 2 mL per min.

As shown in Figure 1A, the presence of four hydroxyl groups (-OH) (3, 5, 7, 4′) in the structure of KAE is the reason why it exhibits a strong antioxidant effect. To confirm the activity of kaempferol (KAE) for 5-HT_3A_ receptors, serotonin and KAE were applied to oocytes expressing human 5-HT_3A_. Receptor expression was confirmed by applying a control ligand serotonin. The induced-inward current (I_5-HT_) was measured after treatment with serotonin or KAE through the two-electrode voltage clamp (TEVC). In Figure 1B, serotonin and KAE 100 μM were applied to confirm the activation manner on 5-HT_3A_. It was confirmed that KAE had a reversible inhibitory manner to 5-HT_3A_ receptors. The inhibition percentage by KAE at 100 μM was 74.6 ± 9.5%. Figure 1C confirms the inhibitory effect of KAE on 5-HT_3A_. Different concentrations (3 μM to 300 μM) of KAE and a fixed concentration of serotonin were used and confirmed that when increasing KAE concentration, the inhibition percentage also increased. KAE inhibited 5-HT_3A_ in a concentration-dependent manner. Figure 1D, using the Hill equation, expressed concentration-dependent manner as a normalized curve fitting, the IC_50_ value was 12.8 ± 2.0 μM and Hill coefficient value was 0.9 ± 0.1. It confirmed that the KAE has the concentration-dependent and reversible manner on human 5-HT_3A_ receptors.

### 2.2. Kaempferol Inhibits Human 5-HT_3A_ Receptor in a Voltage-Independent and Non-Competitive Manner

**Figure 2:**
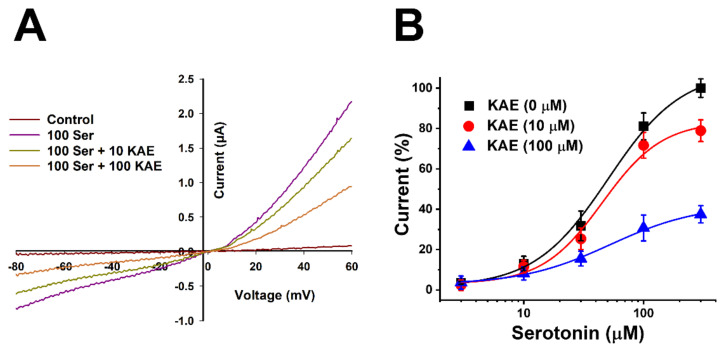
(**A**) Effects of voltage on the relationship of KAE with human 5-HT_3A_ receptor. Different voltages ranging from −80 mV to +60 mV were applied to oocytes injected with water (control) or 5-HT_3A_ mRNA using the voltage clamp ramp protocol. The holding potential of the membrane was fixed at −80 mV. The color of each line indicates the applied condition (red = water injection, purple = 100 μM serotonin applied, green = 100 μM serotonin and 10 μM KAE applied, orange = 100 μM serotonin and 100 μM KAE applied; *n* = 8–10 oocytes from four different frogs). (**B**) Confirmation of a non-competitive interaction between KAE and human 5-HT3A. In oocytes injected with human 5-HT_3A_ mRNA, the concentration of KAE was fixed at 0 μM (■), 10 μM (●), or 100 μM (▲) while the concentration of serotonin ranged from 1 to 300 μM. Membrane holding potential was fixed at −80 mV. Each point represents the current percentage and error value due to change in the concentration of serotonin. Data are presented as mean ± SEM (*n* = 7–9 oocytes from four different frogs) according to statistical analysis.

To confirm whether the inhibition manner of the 5-HT_3A_ receptor by KAE was affected by voltage, the clamp voltage was artificially changed from −80 to +60 mV. Results are shown in Figure 2A. Water-injected oocytes (red line) were not affected by KAE. After applying 100 μM of serotonin to 5-HT_3A_ mRNA injected oocytes (purple line), the constant slope was not affected by voltage fluctuation and the reversal potential was close to 0 mV. There was only a difference in induced-inward current between treatments with 10 μM (green line) and 100 μM (orange line) of KAE. Results confirmed that KAE inhibition was not affected by voltage fluctuations. Thus, it had an inhibitory effect in a voltage-independent manner. To determine whether KAE had a competitive manner to inhibit the 5-HT_3A_ receptor, KAE concentration was fixed while serotonin concentration was changed. After fixing the concentration of KAE to be 0 μM (■), 10 μM (●), or 100 μM (▲), serotonin concentration was changed from 1 μM to 300 μM. Results are presented as sigmoid curve expectation through the Hill equation. As shown in Figure 2B, efficacy change was confirmed rather than potency. KAE showed an inhibitory effect in a non-competitive manner. When applied KAE concentrations were 0 μM, 10 μM, and 100 μM, effectiveness indicator E_max_ values were 100.4 ± 2.1%, 79.3 ± 4.8%, and 37.7 ± 1.2%, respectively.

### 2.3. D Docking Modeling of Human 5-HT_3A_ Receptor Interacting with Kaempferol

**Figure 3:**
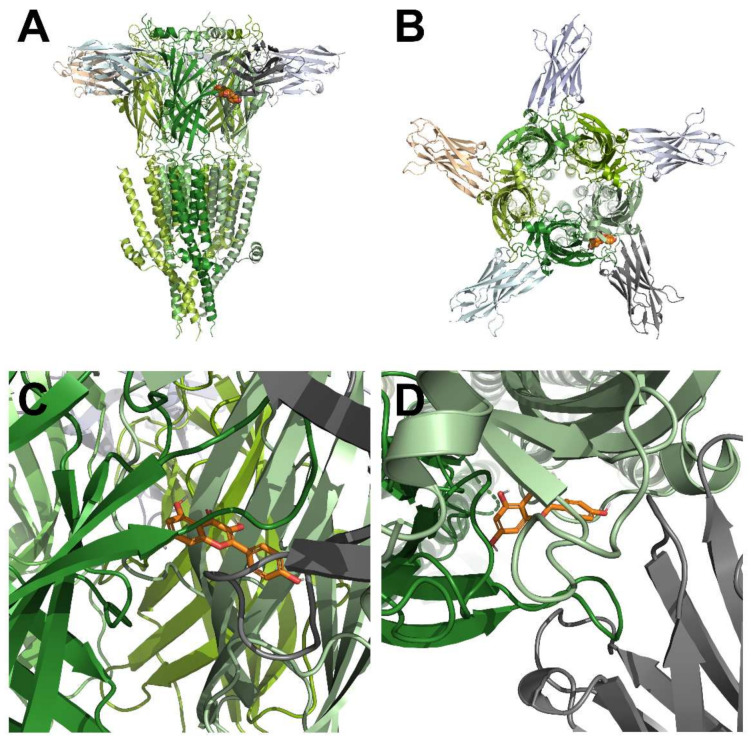
3D docking modeling of KAE on human 5-HT_3A_ receptor. (**A**–**C**) Docked complex of 5-HT_3A_ receptor and KAE from the side. (**B**–**D**) Docked complex of 5-HT_3A_ receptor and KAE from the top.

**Figure 4:**
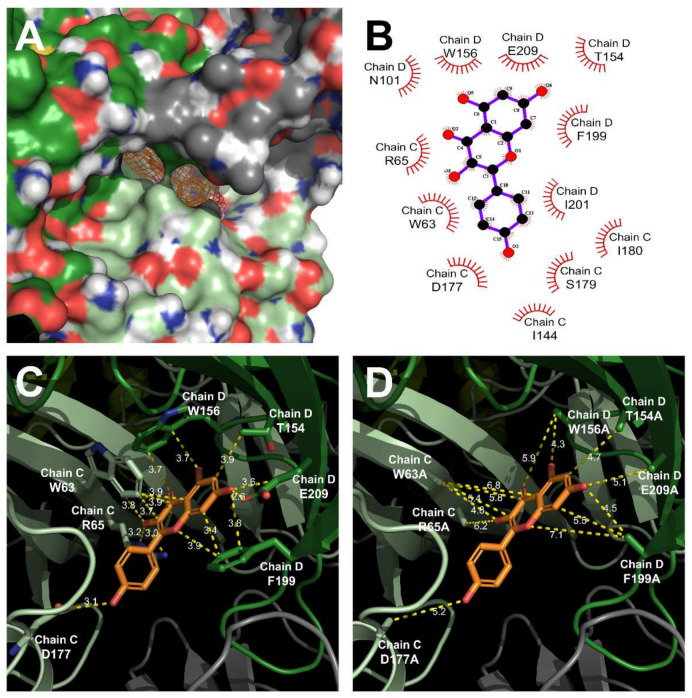
KAE binding pocket and docking modeling of human 5-HT_3A_ receptor by 3D protein modeling. (**A**) 5-HT_3A_ protein surface modeling on KAE binding site. KAE is docked between the two A subunits of the extracellular domain (ECD). (**B**) Interaction residues of KAE in 5-HT_3A_ are predicted by 2D schema. (**C**) Interaction energy of KAE between the two A subunits of the wild-type human 5-HT_3A_ receptor. (**D**) Interaction energy of KAE between the two A subunits of the mutant-type human 5-HT_3A_ receptor. Distances between each chain, residue, and atom are shown in the figure.

Figure 3 shows the KAE binding site on the human 5-HT_3A_ receptor through the protein modeling. It is known that 5-HT_3A_ has a homo pentamer structure composed of five A subunits and three domains: an extracellular domain (ECD), which is the main binding site for ligand and competitive antagonist, a transmembrane domain (TMD), which is the main binding site for non-competitive antagonists with ions conductive structure, and an intracellular domain (ICD) [22]. Each subunit of TMD is composed of four α helices (M1–M4). The M2 domain faces the ion pore [23]. In Figure 4, to confirm the interaction site of KAE on the human 5-HT_3A_ receptor, the in-silico protein-ligand complex modeling was conducted to determine their interaction residues. Figure 4A shows the KAE binding interaction in the protein surface model and Figure 4B shows the positions of residues that are likely to affect KAE binding. To consider the binding pocket in the most stable energy state, the interaction with KAE was implemented as a 3D model based on the crystal structure information of 5-HT_3A_ (PDB ID: 6Y1Z). Interaction distances of KAE with each residue of wild-type and mutant-type 5-HT_3A_ receptors are shown in Figure 4C,D. The binding site of KAE is located at the A + A interaction site adjacent to the two A subunits of ECD. This position shared W63, R65, N101, F199, and E209 residues of the conserved aromatic cage of the serotonin binding site [8]. The two adjacent A subunits and chains are shown in different colors. The distance between atoms is also shown in the Figure 4.

### 2.4. Inhibitory Effect of Kaempferol on a Double-Mutant Type Human 5-HT_3A_ Receptor

**Figure 5:**
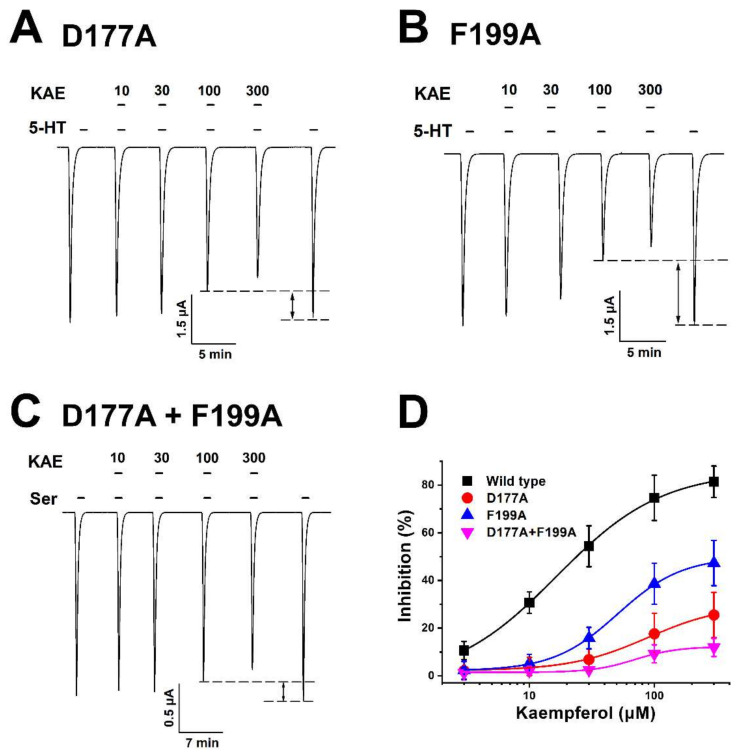
Inhibition of KAE according to mutant-type of 5-HT_3A_ receptor. (**A**–**C**) Inward current when KAE was applied to 5-HT_3A_ single-mutant types (D177 or F199 residue substitution) and double-mutant type (both D177 and F199 residue substitutions). The applied concentration of serotonin was fixed at 100 µM, while the concentration of KAE was 10 µM, 30 µM, 100 µM, or 300 µM. Black bar indicates the applied serotonin and KAE. The black dot line indicated in the figure represents the inward current changed when KAE was applied with the control ligand (5-HT, 100 μM) (*n* = 6–8 oocytes from four different frogs). (**D**) Inhibition percentage of wild-type and single or double-mutant type of 5-HT_3A_ receptor by KAE. Each point represents the inhibition percentage and error value for each concentration of KAE. Data are presented as mean ± SEM (*n* = 9–11 oocytes from four different frogs) according to statistical analysis.

Among KAE’s eight interaction sites (Figure 3B) identified in the human 5-HT_3A_ receptor, a point mutation was performed to determine which residues could critically affect their binding. Each candidate residue was substituted with alanine in the mutagenesis experiment. After mRNA was injected into oocytes and expressed, the activity of the 5-HT_3A_ receptor was measured with TEVC. It was expected that residues that did not affect the binding of KAE would not significantly affect the binding if they were substituted with alanine. The most influential interaction residues were identified by confirming the inhibition of wild-type and mutant-type 5-HT_3A_ by KAE. Results are shown in Figure 5A,B. To confirm that the residue significantly affected the KAE’s inhibition ability, each mutant type, and the inhibition percentage of KAE, were presented as a sigmoid curve using Hill equation. Results are shown in Figure 5D. The half-inhibitory concentration (IC_50_) of KAE was 23.4 ± 23.9 µM for wild-type 5-HT_3A_, 41.4 ± 7.4 µM for D177 residue mutant, and 57.2 ± 20.8 µM for F199 residue mutant. Maximum-inhibitory percentage (I_max_) by KEA was 79.3 ± 4.5% for wild-type 5-HT_3A_, 47.2 ± 1.3% for D177 residue mutant, and 25.8 ± 1.42% for F199 residue mutant. In the case of D177 and F199 double-mutant, the IC_50_ value was 26.0 ± 9.5 μM and the I_max_ was 11.9 ± 1.2%. The substitution of D177 and F199 residues of 5-HT_3A_ receptor with alanine significantly offset the inhibition by KAE (Figure 5). These results confirmed that the crucial binding sites of human 5-HT_3A_ for KAE were D177 and F199. Table 1 shows KAE inhibition according to 5-HT_3A_ mutant-type obtained by point mutation, after selecting the most promising candidate residues by predicting the interaction between wild-type or mutant-type 5-HT_3A,_ and KAE and I_max_ (maximum-inhibitory percentage), IC_50_ (half-inhibitory concentration), and n_H_ (Hill coefficient) values are shown.

## 3. Discussion

Kaempferol (KAE) has a strong antioxidant effect due to the presence of a C2-C3 double bond, a C4 oxo group, and four hydroxy groups (-OH) (3, 5, 7, 4’) in its structure [15]. Reactive oxygen species (ROS) scavenging is a very important function. Excess ROS can induce oxidative stress and provoke cellular redox change, leading to mitochondrial dysfunction, and because of this, the apoptosis-related factors are released into the cytosol, which can lead to programmed cell death [24]. Brain cells’ excessive ROS can react with macromolecular cells through oxidation, leading to cell death or necrosis [25]. Through an in vivo test, it has been confirmed that mice fed with KAE show improvement of arrangement, distribution, and morphological structure of neuronal cells in their brains [26]. In addition, it has been confirmed that KAE can decrease oxidative stress by regulating the expression of apoptosis-related protein [26]. KAE has antioxidant effects in the CNS. It can prevent apoptosis caused by ROS stress. Thus, KAE seems to have a neuroprotective function.

This study confirmed the interaction between 5-HT_3A_ and KAE at the molecular level. KAE inhibited 5-HT_3A_ receptors in a concentration-dependent and a voltage-independent manner. Regarding the inhibitory manner of KAE, when applying serotonin, in the case of a competitive antagonist, a changed potency (effective dose), but a non-competitive antagonist has different efficacy (maximum effect) [27,28]. The present study confirmed that KAE possessed an inhibitory effect in a non-competitive manner that affected the efficacy, but not the potency (Figure 2B). The binding site of serotonin is in the A+A subunit interaction site. Other subtypes also have this interaction site, which is the reason why serotonin acts as a ligand in 5-HT_3AB_ and other 5-HT_3_R subtypes. Therefore, it is highly possible that a competitive 5-HT_3_R antagonist can act on other 5-HT_3_R subtypes [23]. In the case of a non-competitive antagonist, it is highly possible that it does not affect other subtypes. Although it is unaffected by the presence of serotonin, it can be a more effective drug against a target receptor than a competitive antagonist [1].

Among the residues that affected the binding of KAE, it was confirmed that D177 and F199 were major binding sites according to changed interaction distances obtained by amino acid residue substitution on the mutant-type 5-HT_3A_ receptor (Figure 5C). Among binding sites, F199 shared with serotonin’s binding pocket residue. However, it was confirmed that D177 had more contribution to the binding through comparison of Imax values. The binding site of KAE in 5-HT_3A_ is in the ECD, which has a major ligand and competitive antagonist binding site at the 5-HT_3A_ receptor and differs from TMD in that it has a major non-competitive antagonist binding site [4].

5-HT_3A_ is mainly expressed in neuronal cells in regions such as the cerebral cortex, hippocampus, amygdala, and olfactory bulb in the CNS. It is also expressed in non-neuronal cells such as T cells, monocytes, synovial tissue, and primary chondrocytes [29]. Its antagonist can be used as a therapeutic agent for physiological, emotional, cognitive, appetite, sleep, sexual, anxiety, learning, and memory dysfunctions [30]. It has been confirmed through in vivo test that excessive activity of 5-HT_3_R can induce excitotoxic neuronal death, leading to early neuronal cell death [31].

A previously study has confirmed the interaction between human 5-HT_3A_ and antagonist Schisandrin C and discussed their relationship with irritable bowel syndrome (IBS) and the potential of Schisandrin C as a therapeutic agent [32]. The KAE used in this study has lipophilia properties and ability to penetrate the lipid bilayer. Thus, it can cross the blood-brain barrier (BBB) [15]. Among 5-HT_3A_ antagonists, those that can cross the BBB are suitable for treating neurological diseases such as memory and cognitive impairment, through their interaction mechanism in the CNS, while agents that cannot pass BBB are suitable for treating IBS, nausea, and vomiting [23]. We confirmed that KAE has a great potential as an antagonist of the 5-HT_3A_ receptor through molecular studies.

5-HT_3_Rs are mostly found in the pre-synaptic region associated with axons and nerve terminals except for the hippocampus [33]. 5-HT_3A_ at the nerve ending can induce rapid Ca^2+^ influx and regulate the secretion of other neurotransmitters (dopamine, acetylcholine, glutamate, GABA, and serotonin) [7]. The main expression regions of 5-HT_3A_ are the neocortex, hippocampus, and subpopulation of GABAergic interneurons of amygdala [33]. The amygdala and hippocampus are regions related to memory, spatial exploration, emotion regulation, and anxiety. On this path, cholinergic and serotonergic pathways mediate memory activation. It has been shown that long-term potentiation (LTP) related synaptic plasticity can be inhibited by 5-HT_3A_ agonist but activated by antagonist [22]. This is due to activation of the serotonergic pathway in the GABA-ergic interneuron. Thus, it can be seen that this is related to the release of other neurotransmitters by 5-HT_3A_ in the mechanisms related to memory and cognition [22].

Alzheimer’s disease is a representative brain disease. It appears to be due to a decrease in cholinergic signaling molecules associated with aging [2]. Age-related cognitive loss is related to dysfunction of serotonergic and cholinergic pathways. Interaction of these two mechanisms is important for the formation of memory and learning [2]. 5-HT_3_R is also expressed in cholinergic axon terminals of the cerebral cortex. It can regulate the release of acetylcholine (ACh) in a tonic inhibition manner. It has been shown that ondansetron, a representative 5-HT_3_R antagonist, can enhance the release of ACh [34]. Comparing the potency of the most representative 5-HT_3_ antagonist, ondansetron and KAE, IC_50_ values were 4.9 nM and 12.8 uM, respectively. Comparing the efficacy of antagonists against *I*_5-HT(100uM)_ of 5-HT_3A_, KAE is 79.3 ± 4.6%, and ondansetron is close to 100% [35,36,37]. In addition, KAE showed non-competitive inhibition through the above study and ondansetron is a competitive antagonist [1]. Therefore, it can be used to treat cognitive impairment and memory loss by blocking serotonergic activity [38].

Parkinson’s disease is caused by a decrease in dopaminergic neurons [19] and can regulate dopamine release through the serotonergic mechanism. It has been reported that decreased serotonin can induce the release of dopamine [39]. KAE is known as an antioxidant effect. In Parkinson’s disease, KAE treatment can increase the amount of dopamine precursor and its metabolites. It can also increase activities of antioxidant related enzymes such as superoxide dismutase (SOD) and glutathione peroxidase (GSH-Px) [19]. These results indicate that KAE can have a synergic effect because KAE not only can regulate serotonergic pathways, but also can treat brain-related diseases caused by excessive ROS. The finding that KAE can inhibit 5-HT_3A_ in the CNS is significant. KAE is expected to have various neuroprotective effects, such as enhancing synaptic plasticity and preventing neurodegenerative diseases (such as Alzheimer’s disease, memory disorder, depression, anxiety, drug abuse, stroke, and seizure), by regulating the release of other signaling molecules through its antioxidant effect.

5-HT_3A_ is closely related to inflammatory responses. 5-HT_3A_ is expressed in extra-neuronal cells, and immune cells (T cells, monocytes, chondrocytes, synovial tissue). The movement of Na^+^ and K^+^ ions through this receptor plays an important role in regulating the activation and carrying out the aggregation of immune cells in inflammatory events [29]. Aggregated immune cells release growth factors and cytokines. ROS and nitrogen generated in this process are closely related to cellular apoptosis. Excessive ROS can induce oxidative stress, which can damage DNA, protein, and lipids [15]. Chronic inflammation can cause diseases such as cancer, asthma, neurological disorders, and Alzheimer’s disease [15]. Excessive activity of 5-HT_3_R can accumulate Ca^2+^, induce ROS generation, promote abnormal glutamate release, cause imbalance of mitochondria membrane potential, and lead to cytochrome c being released into the cytosol, which can induce apoptosis (caspase pathway) [38,40]. Therefore, anti-inflammatory properties using 5-HT_3A_ antagonists can prevent neuronal apoptosis caused by excessive immune activation, thus preventing neuronal loss with a neuroprotective effect.

5-HT_3_R has subtypes A-E [41]. Studies on subtypes 3A, 3B, and 3AB are actively being carried out. Studies on C, D, and E subtypes are also in progress. Antagonists of 5-HT_3_R have been used to treat vomiting and nausea. However, these 5-HT_3_R receptors regulate various regions of the CNS in a complex manner and modulate the release of other neurotransmitters. They are also involved in nerve interactions. Thus, effects that can be obtained by controlling these receptors are vast [23]. Since 5-HT_3_R regulates the release of other neurotransmitters at synaptic terminals, this mechanism can be targeted as a good approach for treating diseases complexly mediated by neurotransmitters. In addition, in cholinergic signaling, increasing the release of acetylcholine by inhibiting 5-HT_3_R can lead to recovery of cognitive function [2]. Thus, the 5-HT_3A_ antagonist KAE has the potential to be used as a therapeutic agent for cholinergic neurotransmitter deficit. Results of this study obtained at molecular levels are highly likely to be verified through pre-clinic stage studies.

## 4. Materials and Methods

### 4.1. Materials

All reagent stocks used for two-electrode voltage clamp (TEVC) experiments were purchased from Sigma-Aldrich (St. Louis, MO, USA). Human 5-HT_3A_ (GenBank accession No.: BC004453) DNA was obtained from OriGene (Rockville, MD, USA). Dimethyl sulfoxide (DMSO) was used to make the agent stocks and the final concentration did not exceed 0.01%. Kaempferol was purchased from Wuhan ChemFac Biochemical (Hubei, China), and 98% pure Kaempferol 3,4,7-triacetate was used.

### 4.2. In Vitro Transcription: Human 5-HT_3A_ Receptor

Human 5-HT_3A_ receptor cDNA was linearized using restriction enzyme XhoI. In vitro transcription was performed using an mMESSAGE mMACHINE T7 Transcription Kit (Thermo Fisher Scientific, Waltham, MA, USA). The final mRNA product was dissolved in 1X Diethyl Pyrocarbonate (RNase-free water) at a final concentration of 1 μg/μL and kept in a deep-freezer at −80 °C until used.

### 4.3. Xenopus Oocytes Preparation and mRNA Microinjection

*Xenopus* oocytes were used in this study as cells for expression of human 5-HT_3A_ receptor. All female frogs used in this experiment were obtained from the Korean Xenopus Resource Center for Research (KXRCR000001). Xenopus management followed the guideline of Chonnam National University institution guidelines (CNU IACUC-YB-2016-07, July 2016). *Xenopus* oocytes were extracted during ice incubation state and isolated as single oocytes using collagenase II (2 mg/mL) in OR2 buffer (82.5 mM NaCl, 2 mM KCl, 1 mM MgCl_2_, 5 mM HEPES, pH 7.4). Isolated oocytes were incubated with ND96 incubation buffer (96 mM NaCl, 2 mM KCl, 1 mM MgCl_2_, 1.8 mM CaCl_2_, 5 mM HEPES, 2.5 mM sodium pyruvate, and 50 mg/mL gentamycin, pH 7.4) at 18 °C until the experiment was performed. Human 5-HT_3A_ receptor mRNA was injected into oocytes using a nanoinjector (Drummond Scientific, Broomall, PA, USA) with a mineral-filled 10 μL microdispenser (VWR, Piscataway, NJ, USA).

### 4.4. Electrophysiology Studies: Two-Electrode Voltage Clamp Data Recording

To confirm the KAE activity on the human 5-HT_3A_ receptor, a two-electrode voltage clamp (OC-725C; Warner Instruments, Hamden, CT, USA) with a Digitata 1550A was used. For the experiment, induced-inward current was converted to digital data with pClamp 10 software (Axon Instruments, Union City, CA, USA). All experiments were performed at room temperature using an oocyte clamp equipped with Digidata. The membrane holding potential was −80 mV.

The voltage relationship experiment for confirming the relationship between induce-inward current and voltage, the voltage was varied from −80 mV to +60 mV using a ramp protocol. Each microelectrode was filled with 3 M KCl having a resistance of 0.2 MΩ. Serotonin and KAE applied in the experiment were diluted in ND96 bath solution (96 mM NaCl, 2 mM KCl, 1 mM MgCl_2_, 1.8 mM CaCl_2_, and 5 mM HEPES, pH 7.5) to specified concentration. The rate of application of the reagent was 2 mL per minute. All recording data were analyzed using Clampfit 9.0 (Molecular Devices, San Jose, CA, USA).

### 4.5. Point Mutation—Site-Directed Mutagenesis: Mutant Gene Amplification

Point mutation was performed using Pfu DNA polymerase (Quikchange II Site-Directed Mutagenesis Kit). Chimeric primer design for PCR was performed under appropriate primer design guidelines. To remove methylated cDNA, Dpn I was used. Transformation of the mutated DNA used pGEM vector. pDNA was purified using a miniprep protocol. The acquired mutated human 5-HT_3A_ pDNA was subjected to plasmid DNA sequencing by Cosmo Gentech Inc (Seoul, Seongdong-gu, Korea) to confirm the target residue mutation.

### 4.6. Molecular Docking Studies: Protein-Ligand Interaction

A molecular docking study was performed using Autodock Tools version 1.5.6 (The Scripps Research Institute, La Jolla, CA, U.S.A.) to confirm the interaction of the human 5-HT_3A_ receptor with serotonin and KAE. The crystal structure was obtained from Protein Data Bank (PDB ID: 6Y1Z, 2.82-Å resolution) for human 5-HT_3A_ receptor modeling. Structures of serotonin and KAE were obtained from PubChem (PubChem IDs: 443027 and 5280863, respectively). Protein-ligand binding modeling was set up in consideration of protein crystal structure, inhibition constant, intramolecular energy, and minimal binding energy using Autodock Tool version 4.2.6 (The Scripps Research Institute, La Jolla, CA, USA). The implemented complex was analyzed with Ligplot ver. 4.5.3 provided by EMBL-EBI and PyMOL ver. 1.8.4.2 provided by Schrödinger. Using PyMOL, 3D docking models of human 5-HT_3A_ receptor wild-type and mutant and ligand complexes were composed and distances between atoms of wild-type and mutant-type 5-HT_3A_ were measured.

### 4.7. Statistical Analysis

All presented experiments were performed at least three times for scientific verification. Data are presented as mean ± S.E.M (standard error of the mean). Induced-inward current data were analyzed with SigmaPlot 10.0 software (Systat Software, Inc., San Jose, CA, USA). To prepare a sigmoid prediction curve for induced peak data, Hill equation reflecting the binding to ligand and macromolecule was obtained with an OriginPro 9.0 software (Origin, MA, USA). The formula used was y = V_min_ + (V_max_ − V_min_) × [x]^n^/([IC_50_]^n^ + [x]^n^), where V_min_ was the minimum current, V_max_ was the maximum current, IC_50_ was the maximum half inhibitory effect percentage of inhibitory activation by KAE, [x] was the concentration of serotonin or KAE, and n was the interaction-coefficient. Statistical significance was considered when *p*-value was less than or equal to 0.05 (*p* ≤ 0.05).

## Figures and Tables

**Table 1:** Effects of KAE on wild-type and mutant of human 5-HT_3A_ receptor.

Mutant Type	V_max_	IC_50_	*n*
Wild type	79.3 ± 4.6	23.4 ± 8.3	1.2 ± 0.3
R65A	82.6 ± 7.4	44.7 ± 9.1	1.8 ± 0.7
W63A	76.3 ± 10.7	33.7 ± 5.7	1.3 ± 0.5
N101A	83.7 ± 8.6	56.4 ± 9.7	1.1 ± 0.6
I144A	71.6 ± 11.8	34.9 ± 11.2	1.5 ± 0.4
T154A	73.5 ± 9.4	33.4 ± 7.9	1.1 ± 0.4
D177A	23.3 ± 13.4	13.9 ± 4.2	1.3 ± 0.2
I180A	89.5 ± 12.1	45.5 ± 12.9	1.8 ± 0.6
F199A	53.3 ± 10.4	21.5 ± 5.7	1.4 ± 0.4
E209A	81.1 ± 9.7	55.1 ± 5.8	1.7 ± 0.6
I210A	78.4 ± 11.4	35.4 ± 9.7	1.8 ± 0.5
D177A+F199A	11.9 ± 1.2	42.9 ± 10.2	1.3 ± 0.4

The values represent the mean ± S.E.M (*n* = 9–11 oocytes from four different frogs). Currents were elicited at a holding potential of −80 mV. IC_50_ (μM), Hill coefficient (n), and V_max_ (%) were determined as described in Materials and Methods.

## Data Availability

Data is contained within the article.
